# Toward a Gender-Sensitive Approach of Psychiatric Rehabilitation in Autism Spectrum Disorder (ASD): A Systematic Review of Women Needs in the Domains of Romantic Relationships and Reproductive Health

**DOI:** 10.3389/fpsyt.2021.630029

**Published:** 2021-04-28

**Authors:** Marine Dubreucq, Julien Dubreucq

**Affiliations:** ^1^Centre référent de réhabilitation psychosociale et de Remédiation Cognitive (C3R), Centre Hospitalier Alpes Isère, Grenoble, France; ^2^Fondation FondaMental, Créteil, France; ^3^Centre de Neurosciences Cognitive, UMR 5229, CNRS & Université Lyon 1, Lyon, France; ^4^Réseau Handicap Psychique, Grenoble, France

**Keywords:** autism spectrum disorder, women, gender-diverse, unmet needs, psychiatric rehabilitation

## Abstract

Later age of diagnosis, better expressive behaviors, increased use of camouflage strategies but also increased psychiatric symptoms, more unmet needs, and a general lower quality of life are characteristics often associated with female gender in autism spectrum disorder (ASD). Psychiatric rehabilitation has shown small to moderate effectiveness in improving patients' outcomes in ASD. Few gender differences have been found in the response to psychiatric rehabilitation. This might be related to the predominance of males in research samples, but also to the lack of programs directly addressing women's unmet needs. The objectives of the present paper were: (i) to review the needs for care of autistic women in romantic relationships and reproductive health; (ii) to review the existing psychosocial treatments in these domains; and (iii) to evaluate the strengths and limitations of the current body of evidence to guide future research. A systematic electronic database search (PubMed and PsycINFO), following PRISMA guidelines, was conducted on autistic women's needs for care relating to psychiatric rehabilitation in romantic relationships and reproductive health. Out of 27 articles, 22 reported on romantic relationships and 16 used a quantitative design. Most studies were cross-sectional (*n* = 21) and conducted in North America or Europe. Eight studies reported on interventions addressing romantic relationships; no published study reported on interventions on reproductive health or parenting. Most interventions did not include gender-sensitive content (i.e., gender variance and gender-related social norms, roles, and expectations). Autistic women and autistic gender-diverse individuals may face unique challenges in the domains of romantic relationships and reproductive health (high levels of stigma, high risk of sexual abuse, increased psychiatric symptoms, and more unmet needs). We discussed the potential implications for improving women's access to psychiatric and psychosocial treatment, for designing gender-sensitive recovery-oriented interventions, and for future research.

## Introduction

Increasing research interest on potential sex/gender differences in autism spectrum disorder (ASD) has led to the description of a “female phenotype” of ASD characterized by similarities in core ASD symptoms (i.e., lifelong social impairment, communication deficits, and repetitive behavior), better expressive behaviors (e.g., sharing interests or more vivid gestures), increased use of camouflage strategies, later age of diagnosis, but also higher increased depression and anxiety, and a general lower quality of life (1). In the present study, we used the term *sex* to refer to biological characteristics and the term *gender* to refer to sociocultural norms, roles, expressions, and expectations (2). Given the high frequency of gender variance (i.e., gender identity or gender expression that does not conform to masculine or feminine gender norms) (3) in ASD, we used self-reported gender identity (i.e., cisgender women, but also transgender or non-binary women) to define female gender in this study (4).

Psychiatric rehabilitation is a person-centered approach that aims to help people with serious mental illness (SMI) or ASD to “be successful and satisfied in the living, working, learning, and social environments of their choice” (5). Therapeutic tools are selected based on people's strengths, weaknesses, needs for care, and personal life goals as part of a customized recovery-oriented action plan (6, 7). The action plan can include strength-based case management, improvements in physical and mental health, peer support interventions, joint crisis plans, cognitive remediation (CR), cognitive behavior therapy (CBT), social skills training (SST), self-stigma reduction, family support, and supported housing and supported employment (SE) (6, 7). Psychiatric rehabilitation interventions have shown small to moderate effectiveness in improving patients'outcomes in adults with ASD (8–11).

Although no gender differences were found in social participation and the employment rates, women were less likely than men to have long-standing friendships and to maintain post-secondary/employment over time (12–14). To date, three studies have looked for potential gender differences in the response to psychosocial treatment in adults with ASD (15, 16). McVey et al. (15) and Visser et al. (17) found no gender differences in treatment outcomes for SST, whereas Sung et al. (16) reported that men benefited more from vocational rehabilitation.

The predominance of males in research samples may induce gender-related biases affecting the development of psychosocial interventions (18). Two recent systematic reviews have reported a large predominance of males in studies on cognitive remediation or social skills training [up to 100% in some studies (8, 19)]. Similar proportions have been reported in studies targeting social skills in dating contexts (20, 21). Autistic women report more unmet needs with respect to their mental health concerns and vocational services (21, 22) and report a higher risk of sexual abuse (×2.2) (23, 24). Gender variance has been associated with higher depression and reduced well-being in autistic women (25). It could be associated with the higher risk of sexual abuse (25). Reproductive and parenting issues, which may be more salient in women with ASD than in men, remain underinvestigated (26, 27).

The objectives of the present paper were: (i) to review the needs for care of autistic women in romantic relationships and reproductive health; (ii) to review the existing psychosocial treatments in these domains; and (iii) to evaluate the strengths and limitations of the current body of evidence to guide future research.

## Materials and Methods

A stepwise systematic literature review (PRISMA guidelines) (28) was conducted by searching PubMed, MEDLINE, and PsycINFO for published peer-reviewed papers using the following keywords: “sexu^*^” OR “romantic relationship” OR “intimate relationship” OR “parent^*^” OR “reproductive health” OR “mother^*^” OR “pregn^*^” AND “women” OR “gender diverse” OR “transgender” OR “non-binary” AND “autism” NOT “valproate” NOT “22q11.” No time restriction was set. Only published papers in English or French were included in the review. The reference list of seven literature reviews on sexuality and autism were screened for additional relevant articles. To be included in this review, the articles had to meet all of the following criteria: (a) have a main focus on women's outcomes; (b) concern a diagnosis of autism spectrum disorder; (c) report on romantic relationships or parenting; and (d) use a quantitative, qualitative, or mixed-methods design. The first author applied the eligibility criteria and screened the records to select the included studies. The last author reviewed each decision. Disputed items were solved through discussion and by reading the paper in detail to reach a final decision. For each study, we extracted the following information: general information (author, year of publication, country, design, population considered, setting, total number of participants, and mean age or age range), outcome measure (scale), the main findings, and the variables relating to quality assessment. Quality assessment was performed using the Mixed Methods Appraisal Tool (MMAT) (29). This tool comprises five sets of criteria for: (a) qualitative, (b) randomized controlled trials, (c) non-randomized trials, (d) descriptive studies, and (e) mixed-methods studies. For the mixed-methods studies, raters assess the qualitative set, the quantitative set, and the mixed-methods set. An overall quality score (low, moderate, high, or very high) was determined based on adequacy in the corresponding set of criteria.

## Results

**Figure 1:**
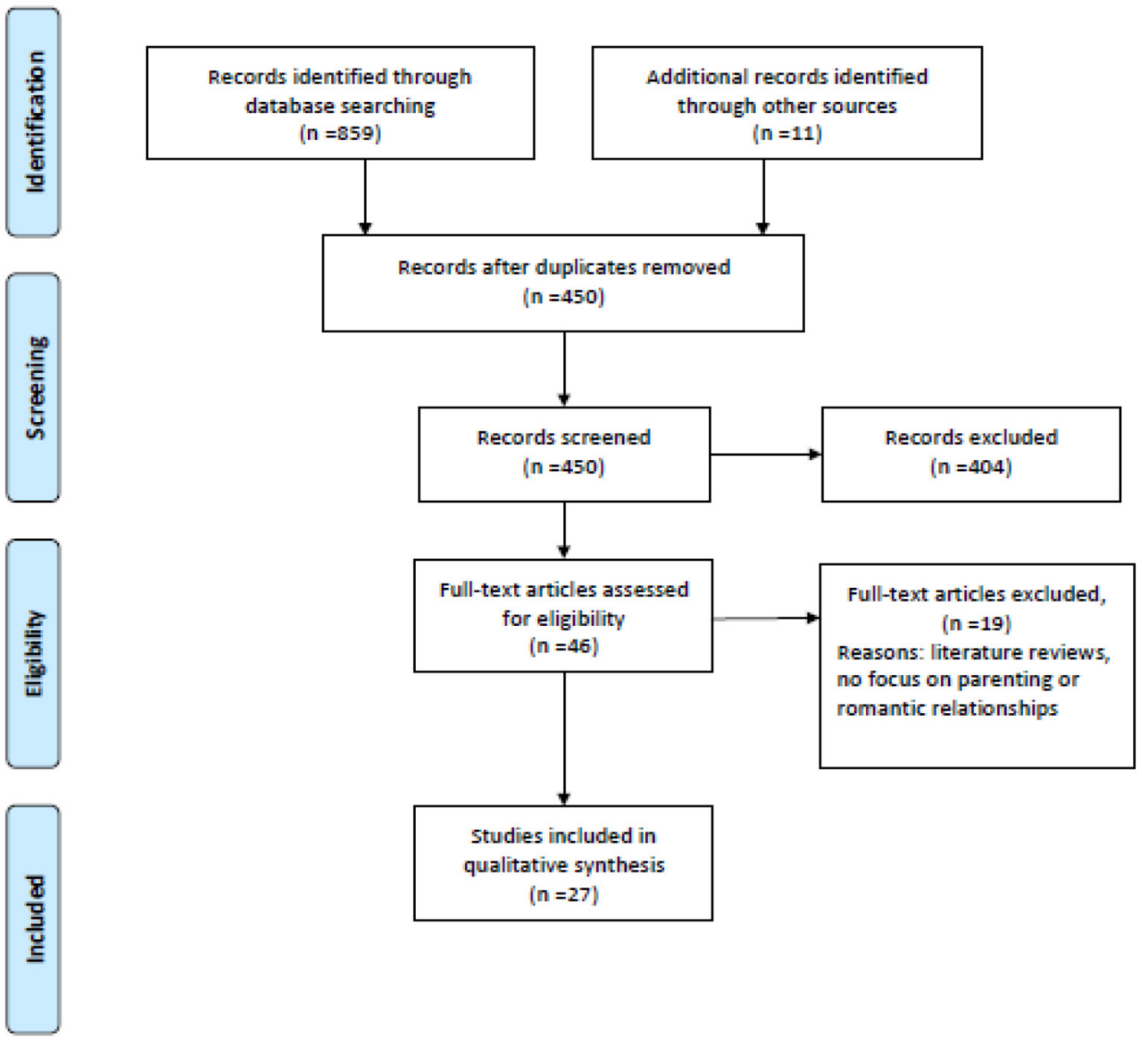
Review process (PRISMA flow diagram).

Our search on January 13, 2021 found 672 articles on PubMed and 187 on PsycINFO. After manually removing all duplicates, there were 450 remaining references. Based on their titles and abstracts, 404 papers were excluded for lack of relevance. Most of these articles focused on other populations (e.g., parents of children or adolescents with autism, healthcare professionals, other neurodevelopmental conditions, and autistic traits in the general public) or other topics (e.g., biological sex differences, physical health, and vocational function). Our search strategy yielded 46 full-text articles. After conducting a full-text analysis of all these papers and excluding those that did not meet the inclusion criteria, we ended up with 27 relevant papers (Figure 1).

The 27 papers included were characterized by the heterogeneity of their samples, methods, and reported outcomes. Most papers reported on romantic relationships (*n* = 22, 81.4%) and used a quantitative design (*n* = 16, 59.3%). Ten studies were qualitative and one was mixed methods. Most studies used cross-sectional designs (*n* = 21, 77.8%). Six studies reported on interventions (22.2%). Two studies were a randomized controlled trials (RCTs) and four studies used quasi-experimental or non-controlled designs. Eleven studies were conducted in North America (40.8%), nine in Europe (33.3%), and seven in Australia (25.9%). Most studies included adult participants (*n* = 22, 81.5%). One study included only adolescents (3.7%) and two studies had mixed samples (7.4%). Three studies (11.1%) included both parents and ASD participants. Of the studies using a quantitative or a mixed-methods design, most reported on sexuality-related outcomes (e.g., sexual desire, sexual awareness, sexual well-being, and sexual assertiveness; *n* = 10, 62.5%). One study reported on social skills in the context of dating (21) and one on general social skills (30). Two quantitative studies used self-reported assessments for parenting-related outcomes (27, 31). The quality ratings of the included studies obtained using MMAT ranged from low to moderate. The results are shown in Table 1.

**Table 1:** Research characteristics of the 27 studies included in the review.

	**Studies about reproductive health/parenting (*N* = 6)**	**Studies about romantic relationships and sexuality (*N* = 22)**	**Total (*N* = 27^a^)**
**Type of study**			
Quantitative study	2 (33.3%)	14 (63.6%)	16 (59.3%)
Qualitative study/case study	4 (66.7%)	7 (31.8%)	10 (37.0%)
Mixed method	0 (0%)	1 (4.6%)	1 (3.7%)
**Design**			
Cross-sectional	6 (100%)	16 (72.7%)	21 (77.8%)
Longitudinal	0 (0%)	6 (27.3%)	6 (22.2%)
**Interventions**	0 (0%)	6 (27.3%)	6 (22.2%)
RCT	0 (0%)	2 (9.0%)	2 (7.4%)
Quasi-experimental	0 (0%)	1 (4.6%)	1 (3.7%)
Uncontrolled	0 (0%)	3 (13.6%)	3 (11.1%)
**Geographical area**			
North America	2 (33.3%)	9 (40.9%)	11 (40.8%)
Europa	3 (50%)	7 (31.8%)	9 (33.3%)
Australia	1 (16.7%)	6 (27.3%)	7 (25.9%)
**Proportion of women**			
100%	6 (100%)	6 (27.3%)	11 (40.8%)
>50%	0 (0%)	6 (27.3%)	6 (22.2%)
<50%	0 (0%)	7 (31.8%)	7 (25.9%)
NR	0 (0%)	3 (13.6%)	3 (11.1%)
**Age of participants**			
<18	0 (0%)	1 (4.6%)	1 (3.7%)
>18	5 (83.3%)	18 (81.8%)	22 (81.5%)
Studies included people less 18	0 (0%)	2 (9.0%)	2 (7.4%)
NR	1 (16.7%)	1 (4.6%)	2 (7.4%)
**Quality of the studies**			
^*^	3 (50%)	3 (13.6%)	6 (22.2%)
^**^	2 (33.3%)	11 (50%)	12 (44.5%)
^***^	1 (16.7%)	8 (36.4%)	9 (33.3%)
^****^	0 (0%)	0 (0%)	0 (0%)

a *The total is not equal to the sum of the numbers in each category because one article (32) was included in both categories (romantic relationships/reproductive health)*.

* *means low quality*,

** *means moderate quality*,

*** *means high quality*,

**** *means very high quality*.

### Romantic/Intimate Relationships

Loneliness, negative self-perceptions, self-stigma, sensory sensitivity, and impaired social communication related to dating situations have been identified as barriers to romantic relationships in ASD (33). Women with ASD may face unique challenges in their adolescence and during the transition to adulthood, such as decrypting dating situations (e.g., judging subtle social cues in dating situations such as flirting, aggression, or coercion) and adopting assertive behaviors in intimate relationships (34). Compared with non-autistic women, autistic women have less sexual interest, but had more experiences and received more unwanted sexual advances (24, 35, 36). They report lower sexual well-being and have an increased risk of sexual abuse (24, 34, 37). Engagement in sexual behaviors to reduce social exclusion and alcohol use to reduce social anxiety could increase the risk of victimization in women with ASD (24, 32, 34).

This risk might be higher for non-binary or transgender autistic women who may also face unique challenges in romantic relationships (e.g., relating to the intersection of ASD and the gender identity stigma) (25, 33, 38). Bargiela et al. (34) and Kanfiszer et al. (32) reported that, after being diagnosed with ASD, autistic women needed to reframe past negative experiences and to discuss gender-related social roles and expectations. Compared with typically developing women and autistic men, autistic women expressed less desire to live with a partner (32, 39, 40). Those living with a typically developing partner reported social and communications challenges, but also to have resources to deal with them (41). Marital satisfaction was higher for autistic women living with an autistic partner (40). Support from healthcare and social professionals was often described as inadequate (41). Table 2 presents the characteristics of the included studies on romantic relationships.

**Table 2:** Included studies reporting on romantic relationships/sexuality in autistic women.

**References**	**Country**	**Design**	**Intervention**	**Sample participant details** **Sample size description Diagnostic criteria**	**Scale**	**Outcomes/findings**	**Quality**
**Quantitative studies**							
Bejerot and Eriksson (35)	Sweden	Cross-sectional	No	ASD group: 50 adults with intelligence within the normal range (26 males and 24 females) Control group: 53 typically developing controls (28 males and 25 females) Age: 20–47	- Swedish modification of the Bem Sex Role Inventory - Questions on sexuality and gender designed for the purpose of the study	- Higher gender variance and proportion of people reporting to have no experience of romantic relationships in the ASD group - Lower libido in the ASD group	^**^
Busch (36)	USA	Cross-sectional	No	*N* = 248 women with ASD (professional diagnosis or self-identified) *N* = 179 women without ASD Mean age: ASD group: 23.2 (18–30) Control group: 21.8 (18–30)	- Sexual History Questionnaire (SHQ) - Sexual Desire Inventory (SDI) - Sexual Experience Questionnaire (SEQ) (adapted 19-item version) - Sexual Satisfaction Scale for Women (SSSW) - Sexual Awareness Questionnaire (SAQ)	- Participants with ASD reported less sexual desire, fewer sexual behaviors, and less sexual awareness than those without ASD - Comparable sexual satisfaction in the ASD and control group	^***^
Byers et al. (37)	Canada and USA	Cross-sectional	No	*N* = 141 (56 men and 85 women) with HFA/AS (professional diagnosis) 60% in romantic relationship Mean age 39.6 (21–73)	- Global Measure of Sexual Satisfaction (GMSEX) - Self-Esteem Subscale of the Sexuality Scale - Hurlbert Index of Sexual Assertiveness - Sexual Arousability and Sexual Anxiety Inventory - Sexual Desire Inventory - Sexual Activity Questionnaire - Sexual Functioning Questionnaire - Sexual Knowledge Questionnaire. - Sexual Cognitions Checklist (SCC)	Less ASD symptoms are associated with greater sexual well-being for autistic men	^**^
Corona et al. (42)	USA	Uncontrolled	Yes. Six-2h sessions over the course of 3 months.	*N* = 8 adolescent with ASD (2 females and 6 males) +one parent for each adolescent (six mothers, two fathers) Mean age of adolescents: 13.4 (12–16)	- Sexual Behavior Scale (SBS) - Adolescent Knowledge Questionnaire - Parent Questionnaire - Parent Satisfaction Questionnaire	- Higher number of sexuality-related topics discussed between parents and adolescents at post-treatment - No difference in the comfort felt when discussing these topics at post-treatment	^*^
Cunningham et al. (21)	USA	Quasi-experimental	Relationship Enhancement (RE) “Ready for Love”	RE-ASD group *N* = 19 (16 males and 3 females) RE group *N* = 19 (14 males and 5 females) Age>18 (76.3% between 18 and 29)	- Dating and Assertion Questionnaire (DAQ) - EQ - SRS	- Improved parent-report social responsiveness in both groups - No significant difference in treatment effectiveness between the two groups	^**^
Georges and Stokes (25)	Australia	Cross-sectional		ASD group: *N* = 309 (219 females and 90 males) Control group: *N* = 261 (158 females and 103 males) Mean age ASD group: 31.01 Control group: 30.20	- Sell Scale of Sexual Orientation - Gender - Identity/ Gender - Dysphoria - Questionnaire for Adolescents and Adults (GIDYQ-AA) - Depression, Anxiety, and Stress Scale-21 (DASS-21) - Personal Well-being Index scale	- Higher psychiatric comorbidities and reduced well-being in autistic individuals compared to typically developing controls - Higher psychiatric comorbidities and reduced well-being in gender variant autistic individuals compared to other autistic participants	^***^
Hénault (43)	Canada	Uncontrolled	Psychosexual program	NR	- Australian Scale for Asperger's Syndrome - Aberrant Behavior Checkist - Friendship Skills Observation Checklist - Inventaire du fonctionnement sexuel de Derogatis	Improvement in skills related to friendships and intimacy	^*^
Jamison et Schuttler (30)	USA	Uncontrolled	The Girls Night Out Model	*N* = 33 adolescents females (28 with ASD, 2 with learning disability, 3 with learning disability and significant social impairment) *N* = 22 parents Mean age: 15.97 (14–19)	- Social Skill Improvement System (SSIS) - Self Perception Profile for Adolescents (SPPA) - Youth Quality of Life-Research Version (YQoL-R) - satisfaction data from parents, participants, and peers.	- Improvements in self-reported social competence, self-reported internalizing symptoms and quality of life -No significant improvements following intervention on the parent-report social competence scale	^**^
McVey (15)	USA	Randomized controlled trial	PEERS (sixteen 1.5h-session)	*N* = 177 HFA (27 females and 150 males) Experimental group: *N* = 88 Control group: *N* = 89 Age > 18	- SRS parent - SSIS-RS (variant of SSRS) - Empathy quotient - LSAS (social anxiety) - TYASSK: social knowledge - SELSA loneliness scale	No gender differences in treatment effectiveness	^***^
Pecora et al. (24)	Australia	Cross-sectional	No	ASD group: *N* = 231 (135 females and 96 males) (self-reported formal diagnosis of HFA or AS) Control group: *N* = 227 (161 females and 66 males) Mean age: ASD group: 25.13 Control group: 22.16	Sexual Behavior Scale: Version 3 (SBS-III)	- Less sexual interest but more sexual experiences in autistic women compared to non-autistic women - More autistic women reported engaging in sexual behaviors that were later regretted, unwanted, or receiving unwanted sexual advances	^***^
Pecora et al. (45)	Australia	Cross-sectional	No	ASD group: *N* = 134 autistic females Control group: *N* = 161 females Mean age: ASD group: 26.2 (18–56) Control group: 22.0 (18–48)	Sexual Behavior Scale-III (SBS-III)	- Higher gender variance and non-heterosexual orientation in autistic women - More negative sexual experiences in autistic homosexual women compared to heterosexual autistic women - More unwanted sexual experiences in autistic homosexual women compared to non-autistic women	^***^
Strunz et al. (40)	Germany	Cross-sectional	No	*N* = 229 high-functioning adults with ASD (professional diagnosis) (137 females and 92 males) Three groups: Currently in relationship, History of relationship Never been in relationship Mean age: 34.9 (18, 20–41, 43, 46–62)	- Need for Social Support subscale of the Berlin Social Support Scales - Personal Distress subscale of the Interpersonal Reactivity Index (IRI) - Dyadic Adjustment Scale (DAS) - Original questionnaire items that assessed the desire for romantic relationships and the reasons for not being in a romantic relationship	- 73% indicated romantic relationship experience and only 7% had no desire to be in a romantic relationship. - Higher marital satisfaction in people whose partner was also on the ASD spectrum - Reasons for being single: tiredness (65%), fear of not meeting the partner's expectations (61%) and not knowing how to meet someone (57%)	^**^
Turner et al. (63)	Germany	Cross-sectional	No	ASD group: *N* = 96 (40 females and 56 males) (professional diagnosis) Control group: *N* = 96 (39 females and 57 males) Mean age: ASD group: 39.2 Control group: 37.9	- International Index of Erectile Function (IIEF) - Female Sexual Function Index (FSFI) - Sexual Inhibition/Sexual Excitation Scales-Short Form (SIS/SES-SF)	- Higher sexual dysfunctions and reduced libido and less sexual satisfaction in autistic women compared to non-autistic women - No difference in sexual satisfaction between autistic men and autistic women	^***^
Visser et al. (17)	Netherlands	Longitudinal randomized controlled trial	Yes (Tackling Teenage psychosexual training program) 18 weekly individual sessions	Intervention group: *N* = 95 (73 males and 25 females) Waiting-list control condition: *N* = 94 (79 males and 15 females) Full IQ ≥ 85 Mean age: Intervention group: 14.4 (12.1–18.8) Control condition: 14.5 (12.0–18.5)	- Sex Problems scale of the Child Behavior Checklist (CBCL) - Self-report and a parent-reported scale measuring specific ASD-related inappropriate sexual behavior	- Improvements in psychosexual knowledge at post-treatment - Higher treatment effects for younger adolescents - No gender differences in treatment effectiveness	^***^
**Qualitative studies**							
Bargiela et al. (34)	UK	Qualitative	No	*N* = 14 women with ASD (formal diagnosis) IQ>70 Age: 26.7 (22–30)	NA	- Four main themes including “passive to assertive” et “forging an identity as a woman with ASD” - High incidence of sexual abuse (9 of 14 participants) - Difficulties to “read” other people's intentions, and struggle to understand if a man was just being friendly or was sexually attracted to them.	
						- Experiences of peer rejection resulted in higher desire for social acceptance and thus increased risk of sexual abuse - Some reported not having known that they could say “no” when they had wanted to refuse sex or other people's advances. Others reported that they had not known how to say “no” or how to leave a situation until it was too late	
						- Women do not readily identify to gender-related stereotypes and report a sense of loss of identity when trying to meet gender-related social expectations	^**^
Barnett et al. (64)	USA	Qualitative	No	*N* = 24 adults with ASD (13 with a feminine identity, 6 with a masculine identity and 5 gender queer or androgynous) (self-identifies as ASD) Mean age: 37 (18–61)	/	- High frequency of gender variance and non-heterosexual orientation - Concerns: courtship difficulties, sensory sensitivity, difficulties in understanding non-verbal communication, difficulties in showing romantic interest, inappropriate sex education experiences - Strategies: using sensory barriers; planning when and how to have sex; negotiating alternatives to sexual scripts predicated on non-disabled experience; and practicing explicit and intentional communication.	^*^
Dubreucq et al. (44)	France	Qualitative	Yes	*N* = 7 women with ASD (professional diagnosis) Mean age: 37.7 (25–52)	/	- Autistic women reported not to readily identify with gendered social norms, roles and expectations - Autistic women reported the need to forge an integrated identity as women with ASD - Challenges in romantic relationships/sexuality: fear to attract unwanted sexual attention (e.g., sending involuntarily non-verbal cues of seduction during social interactions); decoding dating contexts in general and when personally involved; knowing what is	^**^
						acceptable or not in romantic relationships; learning how to cope with dating situations/unwanted sexual attention; prevention of sexual abuse	
Kanfiszer et al. (32)	UK	Multi-stage narrative analysis	No	*N* = 7 women with ASD (professional diagnosis) Age: 20–59	/	- Autistic women do not readily identify to gender-related social norms, roles and expectations - High frequency of sexual abuse (difficulty to know how to say no to an unwanted relation)	^**^
Sala et al. (33)	Australia	Qualitative	No	ASD group: *N* = 31 (14 females, 10 males and 7 “non-binary”) Control group: *N* = 26 (20 females, 5 males and 1 “non-binary”) Mean age: ASD group: 32.29 (19–54) Control group: 33.1 (20–57)	/	- Most of the enablers/barriers of intimacy were common to both groups - ASD-specific barriers included anticipated stigma, self-stigma, uncertainty about relationships and communication.	^***^
Smith et al. (41)	Australia	Qualitative	No	*N* = 13 persons in neurodiverse relationship Age: 25–>65	/	- Challenges in neurodiverse relationships: communication, difficulties in reading and interpreting emotions - Facilitators: strengths-based roles of each partner; care and support for each other - Inadequacy of the existing counseling service provision	^**^
Strang et al. (38)	USA	Qualitative	Yes	Autistic/neurodiverse gender-diverse (A/ND-GD) youth with gender dysphoria and ASD or social communication disorder (*N* = 31) Mean age: 15.92 (12–19) Parents of A/ND-GD youth (30 mothers and 16 fathers) (N = 46)	/	- Priorities for adolescents: importance of connecting with other gender diverse youth; experiencing a range of gender-diverse role models to make gender exploration and/or gender affirmation more concrete - Priority for parents: the need for ASD- related supports for their children (e.g., social skills training) as well as provision of an ASD-friendly environment that fosters exploration of a range of gender expressions/options.	^***^
**Mixed method**							
Baldwin and Costley (39)	Australia	Cross-sectional Mixed-methods	No	*N* = 282 (82 females and 200 males) with HFA (Professional diagnosis) IQ>70 Mean age: Women group: 32.7 (18–64) Men group: 33.2 (18–70)	- 28 items divided across six main sections, namely, (1) health and well-being, (2) education, (3) employment, (4) social and community activities, (5) support needs and (6) future aspirations.	- 56% of those not already in a relationship included “dating and relationships” as one of the areas in which they would like more support. - 19% of these people listed marriage or a relationship as one of their top three hopes and aspirations for the future. - Females were significantly less likely (19%) than males (40%) to list marriage or a romantic relationship as one of their top three hopes and aspirations for the future	^**^

*AS, Asperger syndrome; ASD, autism syndrome disorder; HFA, high-functioning autism; IQ, intellectual quotient; NA, not applicable; NR, not reported*.

* *means low quality*,

** *means moderate quality*,

*** *means high quality*,

**** *means very high quality*.

Four interventions addressed romantic relationships in women with ASD. One was a general SST program including social skills in dating (Program for the Education and Enrichment of Relationship Skills, PEERS) (15) and the other a social skills in dating program (Ready for Love) (21). Two programs were psychosexual trainings for autistic adolescents [socio-sexual training (43) and Tackling Teenage (17)]. Participants in these interventions were mostly male (>75%) (15, 17, 21). McVey et al. (15) and Visser et al. (17) reported no gender differences in treatment effects for PEERS and Tackling Teenage. One SST intervention was designed for autistic female adolescents (the Girls Night Out model) (30), but did not address dating or romantic relationships. Two qualitative studies assessed stakeholders' needs to design new interventions (38, 44). Common factors to these interventions were the emphasis put on: (i) group-based interventions to receive support from other participants; (ii) peer support; (iii) discussions about gender variance and gender-related social roles and expectations; iv) improving social cognition and social skills; and v) interventions supporting the relatives (38, 43, 44). Descriptions of the interventions are shown on Table 3.

**Table 3:** Interventions targeting romantic relationships/sexuality.

**Name of the intervention**	**Type of intervention**	**Public**	**Topics**	**Main results**
PEERS® for Young Adults (Program for the Education and Enrichment of Relational Skill) (15)	16 –session Group-based Social Skills Training	Adults with ASD (>18 years old)	1. Trading Information and Starting Conversations 2. Trading Information and Maintaining Conversations 3. Finding a Source of Friends 4. Electronic Communication 5. Appropriate Use of Humor 6. Entering Group Conversations 7. Exiting Conversations 8. Get-Togethers 9. Dating Etiquette I: Letting Someone Know You Like Them 10. Dating Etiquette II: Asking Someone on a Date 11. Dating Etiquette III: Going on Dates 12. Dating Etiquette IV: Dating Do's and Don't 13. Handling Disagreements 14. Handling Direct Bullying 15. Handling Indirect Bullying 16. Moving Forward and Graduation	No gender differences in treatment effectiveness
“Ready for Love” (Relationship Enhancement/RE-ASD) (21)	Eight 2-h weekly group sessions Dating social skills program	Adults with ASD (>18 years old)	How to flirt, assessing if someone likes you, and asking someone out on a date	- Improved parent-report social responsiveness in both groups - No significant difference in treatment effectiveness between the two groups
Girls Night Out Model (30)	12–16 2-h weekly group SST sessions	Adolescents females with ASD and peers without ASD	- No session on dating social skills - Content related to social interaction, self-care skills, self-determination and leisure activities	Improvements in self-reported social competence, self-reported internalizing symptoms and quality of life No significant improvements following intervention on the parent-report social competence scale
Tackling Teenage psychosexual training program (17)	18 weekly individual sessions Psychosexual trainings for autistic adolescents	Adolescents with ASD and their parents	Discussing puberty, appearances, first impressions, physical and emotional developments in adolescence, friendships, falling in love and dating, sexuality and sex (e.g., sexual orientation, masturbation, and intercourse), pregnancy, setting and respecting boundaries and safe internet use	Improvements in psychosexual knowledge at post-treatment Higher treatment effects for younger adolescents No gender differences in treatment effectiveness
Asperger's syndrome and sexuality (43)	12 weekly group- sessions Psychosexual trainings for autistic adolescents	Adolescents or adults with high-functioning autism	Love and friendship Sexual intercourse and other behaviors Emotions Sexual diseases and prevention Sexual orientation Alcohol, drugs and sexuality Sexual abuse and inappropriate behaviors Theory of mind, communication and intimacy	Improvement in skills related to friendships and intimacy
Program about sexuality and relationships (42)	Six 2-h weekly group sessions	Adolescents with ASD and their parents	Discussing puberty, intimacy, hygiene, relationships, dating, sexuality and sex (e.g., sexual orientation, masturbation, and intercourse), preventing abuse	Higher number of sexuality-related topics discussed between parents and adolescents at post-treatment No difference in the comfort felt when discussing these topics at post-treatment
Strang et al. (38)	Qualitative study assessing parent and adolescents' needs to design new interventions	Gender diverse autistic adolescents	Need for group-based sessions: - How to deal with issues specific to gender diverse adolescents - Support gender expression/style - Provide gender-diverse role models and gender exploration opportunities - Provide opportunities for group members to chat during the group - Improve executive function and social skills - Learn how to notice and avoid risky situations Need for group-based sessions for relatives too	/
Dubreucq et al. (44)	Qualitative study assessing autistic women' needs to design new interventions Eight 2-h weekly group sessions	Adults women with ASD	Need to discuss gender-related social norms, roles, expectations and pressures Need to discuss gender variance and to forge an integrated identity as a woman with ASD Need to learn about: non-verbal cues of seduction/disinterest, how to avoid unwanted attention, decoding dating contexts and reading the other's intentions, what is acceptable or not in a relationship; taking stock on one's strengths and limitations; how to deal with dating contexts; how to deal with unwanted attention/risky situations	/

### Reproductive Health/Parenting

To date, a few qualitative studies and two quantitative studies have investigated the experiences of motherhood in ASD. Kanfiszer et al. (32) reported that some autistic women identified motherhood as a potential trigger of stress more than a rewarding experience and reported to have “no maternal instinct.” In a sample comparing the experiences of 355 mothers with ASD with those of 132 non-autistic mothers, Pohl et al. (27) have found that autistic mothers had an increased frequency of perinatal depression. Although most autistic mothers described parenting as a rewarding experience, they were more prone than non-autistic mothers to find it also challenging and isolating (27). Autistic mothers were more likely to report parenting difficulties (e.g., with household responsibilities or with the multitasking demands of parenting) and to be dissatisfied with service provision during perinatal care (e.g., feeling judged in their parenting abilities) (27, 46, 47). They described stressful and negative experiences in communicating with healthcare professionals and social workers about their child (27, 46, 47). Some women reported to have concealed their diagnosis to providers to avoid stigmatizing behaviors and child protective services implications (46, 47). Other factors associated with negative experiences in perinatal care included uncertain and stressful environment and sensory sensitivity (46–48). Sundelin et al. (31) have reported that, compared to children of non-autistic mothers, those of autistic mothers had poorer obstetrical and neonatal outcomes (e.g., pre-term birth, preeclampsia, and cesarean delivery). Table 4 shows the characteristics of the included studies on parenting.

**Table 4:** Included studies reporting on reproductive health/parenting in women with autism.

**Quantitative studies**							
**References**	**Country**	**Design**	**Intervention**	**Samples**	**Scale**	**Outcomes/findings**	**Quality**
Pohl et al. (27)	UK	Cross-sectional	No	ASD group: *N* = 355 autistic mothers (professional diagnosis and self-identified as autistic) Control group: *N* = 132 non-autistic mothers	/	Compared to non-autistic mothers, autistic mothers reported: higher frequency of perinatal depression, higher parenting difficulties (e.g., multi-tasking or household responsibilities), more stressful interactions with professionals, more feelings of being misunderstood by professionals, more worries about others judging their parenting and less support in parenting. Motherhood was described more frequently as an isolating experience and less often as a rewarding experience	^**^
				Mean age: ASD group: 42.7 Control group: 44.6		Most of autistic mothers reported however mothering as a rewarding experience	
Sundelin et al. (31)	Sweden	Nationwide population-based cohort study	No	ASD group: *N* = 2,198 births to 1,382 women with autism Control group: *N* = 877,742 births to 503,846 women never diagnosed with autism		Women with autism were at increased risk of preterm birth. Maternal autism was also associated with an increased risk of elective cesarean delivery and preeclampsia	^***^
Donovan (48)	USA	Qualitative	No	*N* = 24 women with ASD Age: NR	/	Three main themes: Having Difficulty Communicating, Feeling Stressed in an Uncertain Environment, and Being an Autistic Mother Challenges: difficulty to communicate with professionals, feelings of being misunderstood (e.g., pain when facial expression was not in adequacy with their internal feelings), fear of child protective services involvement; need for more time before answering and for explicit communication	^*^
Gardner et al. (46)	USA	Qualitative	No	*N* = 8 women with ASD Mean age: 39 (27–52)	/	Four major themes: Processing Sensations, Needing to Have Control, Walking in the Dark, and Motherhood on My Own Terms - Pre-natal needs: taking into account sensory sensitivity; need for direct answers and clear information - Delivery: sensory sensitivity and need for having control on the situation - Post-natal period: feelings of being judged in their parenting by professionals	^*^
Kanfiszer et al. (32)	UK	Qualitative	No	*N* = 7 women with ASD (professional diagnosis) Age: 20–59	/	Motherhood perceived as a “daunting” and stressful experience; diagnosis helpful in reframing past experiences for those who chose not to have children; the “daunting” nature of motherhood is a barrier to start a family.	^**^
Rogers et al. (47)	Australia	Case study	No	*N* = 1 woman with ASD (professional diagnosis) Age: 26 years-old	/	Difficulties to communicate with professionals Sensory sensitivity Feeling of being judged in mothering ability	^*^

* *means low quality*,

** *means moderate quality*,

*** *means high quality*,

**** *means very high quality*.

According to Pohl et al. (27), autistic mothers were dissatisfied of the support they received during perinatal care and felt that they should be offered extra support because of their autism. Gardner et al. (46), Rogers et al. (47), and Pohl et al. (27) reported a need for clear and accurate information about normal pregnancy, potential complications, delivery, and post-partum care. To our knowledge, no published studies described interventions to improve reproductive health outcomes and to support parenting in autistic mothers. In the FondaMental Advanced Center of Expertise for ASD (FACE-ASD) of Grenoble, consultation with a trained midwife has been added to the diagnosis and functional assessment. The objectives were: (i) to assess the desire to become a parent; (ii) to answer questions relating to ASD, pregnancy, and perinatal health outcomes (e.g., concerns about passing on ASD to their child, information about the stay in the maternity ward, cesarean section, circadian rhythms, and lactation); (iii) to propose strengths-based case management and a shared recovery-oriented action plan to improve well-being and pregnancy-related outcomes; (iv) to provide individual and/or family psychoeducation; and (v) to improve the communication with perinatal health professionals and social workers. Actions addressing mothers and their partners' needs or aiming to improve knowledge of perinatal health professionals about ASD and to reduce ASD-related stigma could be provided during the perinatal period (i.e., from preconception to 1 year after childbirth). A group-based program on reproductive health and parenting issues for women with ASD and their partners was also developed. It includes three 2-h sessions during which participants and facilitators discuss issues related to reproductive health and parenting (e.g., concerns about heredity or communication with healthcare professionals). A detailed description of these actions is presented in Table 5.

**Table 5:** Example of interventions to support autistic women in reproductive health (FondaMental Advanced Center of Expertise for ASD (FACE-ASD) center of Grenoble).

	**Individual interventions**	**Group-based interventions**
Interventions toward women with ASD	**Assessment by a trained midwife** - Physical health - Reproductive health history and needs for care - History of childhood/domestic abuse - Conception of gender social norms, roles, expectations and pressures (e.g., gender variance, desire to become mother or not, centrality of mothering…) - Fears about becoming mother (e.g., fears about pregnancy, delivery or post-partum care, being an autistic mother, communication with healthcare and childcare professionals, etc…) **Strengths-based case-management from preconception to post-partum care by a trained midwife** - Assess strengths/Building on strengths - Answer to questions relating to ASD, pregnancy and perinatal health outcomes (e.g., concerns about passing on ASD to their child, information about the stay in the maternity ward, cesarean section, circadian rhythms and lactation, fear of being judged by others on mothering, prevention of perinatal depression) - Shared strengths-based action plan to improve well-being and pregnancy-related outcomes - Making empowered decisions about whether to disclose or not the diagnosis of ASD - Working on how to communicate with perinatal health professionals and social workers - Coping with stigma - Getting support	**Four-session group-based psychoeducation:** - Factors influencing decision-making about becoming mother or not - ASD and perinatal outcomes - Getting support - Making empowered decisions about disclosure
Interventions toward perinatal professionals	- Facilitate the communication between the mother and perinatal health professionals - Provide support to help professionals to meet ASD-related needs for care - Reducing stigma - Prevent unnecessary child protective services involvement	- Awareness and training actions: ASD and perinatal outcomes, screening of perinatal depression, ASD-related needs for care in the maternity ward (e.g., reducing sensory sensitivity issues and improving communication with autistic mothers), stigma-related issues, ASD and parenting

## Discussion

### Main Findings

Altogether, the results of this systematic review may be summarized as follows: (i) women report several unmet needs in the domains of romantic relationships and reproductive health; (ii) psychosocial treatment addressing skills related to romantic relationships remains underdeveloped and is often inadequate to women's needs for care; (iii) research on reproductive health and parenting in autistic mothers is almost inexistent, and there is a lack of interventions supporting future mothers or mothers with ASD; and (iv) stigma reduction could improve women's outcomes in romantic relationships and reproductive health.

### Interpretation of the Results

#### Access to Adequate Healthcare

While there are few gender differences in the pattern of service utilization, women report more unmet needs and more negative experiences with healthcare providers and social workers (22, 27). Compared with autistic men and typically developing women, women with ASD report more often comorbid physical health conditions (e.g., polycystic ovary syndrome, 7.8 *vs*. 3.5% in the general population) (49) and less utilization of women's health services (OR = 0.59, 95% CI = 0.48–0.72) (50). The barriers to accessing adequate healthcare include providers' lack of knowledge and dedicated training regarding autistic women' needs for care and the lack of specific services for adults with ASD (22, 49, 50). Although it has been proposed that autistic women's lower access to women's health services could be explained by less frequent sexual experiences (50), several studies have reported the opposite (23, 24, 34). A higher motivation to engage in social relationships, a higher desire to fit in social contexts and to meet gender-related social expectations, preference for opposite-sex friendships, and increased use of camouflage strategies are characteristics often associated with female gender (32, 34, 51, 52).

#### Camouflaging, Stigma, and Personal Identity: Implications for Psychiatric Rehabilitation

Qualitative research reported that women found social relationships challenging and felt exhaustion or identity loss feelings when using camouflage strategies (32, 34, 51). Camouflaging refers to the use of conscious or unconscious strategies to minimize or mask autistic characteristics during a social interaction (1). Reasons for camouflaging include the desire to fit in a non-autistic world, anticipated stigma, concerns about the impression made when not camouflaging, and self-stigma (53). Cooper et al. (3) have reported that autistic women identified less with their gender group and had lower in-group value (i.e., one's perception of his own group) compared to autistic men and typically developing women. Hull et al. (1) have found an increased use of camouflage strategies in non-binary autistic individuals and women compared to autistic men. The negative effects of camouflaging strategies on psychiatric symptoms (i.e., higher psychological distress, anxiety, and depression) (53) might be more pronounced in this population (1).

SST is a group-based intervention that encompasses a set of behavioral strategies for teaching new skills based on social learning theory (54). Compensation strategies refer to the use of alternative cognitive routes to compensate for ASD-related social-cognitive difficulties (e.g., impairments in theory of mind) (55). They have been associated with higher verbal abilities, preserved executive function, and improved social skills, but also poorer mental health (56, 57). Although it has been proposed that compensation could be an adaptive coping strategy contributing to SST effectiveness on social skills, it may partially overlap with camouflaging (1, 56). SST could increase the use of camouflage strategies and indirectly contribute to increased psychological distress and suicidal ideation (1, 4, 58). To our knowledge, existing SST interventions do not address the distinction between compensation and camouflaging and their respective consequences on treatment outcomes and mental health. Future programs should address this distinction to improve compensation while reducing camouflaging (56, 57). SST also carries the risk of reinforcing common gender stereotypes about how a person should act in a given sociocultural context (4, 59). The inclusion of gender-sensitive content (e.g., discussing gender variance and gender-related social roles, norms, and expectations) in SST programs could prevent the internalization of gender social norms and improve treatment effectiveness in autistic women and gender-diverse individuals (4, 38).

Camouflaging has been related to secrecy coping and could be used to avoid the stigma of being labeled with ASD (60). Experienced stigma, anticipated stigma, and self-stigma were identified as barriers to intimacy in ASD (33). This concurs with findings in serious mental illness where self-stigma has been associated with reduced capacity for intimacy and more negative parenting experiences (61). Self-stigma has been found to affect one in five people with ASD (7, 62). It has been associated with poorer recovery-related outcomes (i.e., reduced self-esteem and well-being and higher depression and suicide risk) (7). Gender-diverse autistic individuals are confronted with multiple sources of stigma (e.g., intersection of ASD and gender identity stigma) and may be at particular risk of self-stigma (33). Future research should further investigate perceived stigma, experienced stigma, anticipated stigma, and self-stigma in gender-diverse autistic individuals. Self-stigma reduction [e.g., narrative enhancement and cognitive therapy (65) or peer-delivered interventions (61)] might improve the clinical and functional outcomes in autistic women, although this remains to be investigated. A longitudinal examination is needed to investigate the potential relationships between camouflage strategies, stigma-related issues, capacity for intimacy, and parenting experiences.

Cage and Troxell-Whitman (53) have found that autistic women were more likely than men to endorse “conventional reasons” for camouflaging (e.g., fitting in university or work contexts). Nagib and Wilton (66) have reported that gender-related assumptions about stereotypical jobs to which they should aspire limited autistic women in their vocational insertions. This might contribute to the lower satisfaction and benefits from vocational support services in autistic women compared with men (16, 22). Future research should investigate whether the integration of gender-sensitive content (i.e., taking into account gender variance and discussing gender-related sociocultural norms, roles, and expectations) in psychiatric rehabilitation (e.g., in cognitive behavior therapy) and vocational support services improves treatment outcomes or not.

#### Reproductive Health and Psychiatric Rehabilitation

Compared to non-autistic mothers, autistic mothers had higher risks of perinatal depression and poorer obstetrical and neonatal outcomes (27, 31). Clinical implications from qualitative research included a need for clear and accurate information (e.g., about normal pregnancy, potential complications, delivery, and post-partum care), for stigma reduction (e.g., reducing the feeling of being judged by perinatal health providers), and for improved self-agency and empowerment (27, 46, 47). Awareness actions toward women's health professionals could improve their understanding of autistic women's needs (e.g., heightened sensory processing and medical examinations) and reduce the stigma associated with autism (27, 46, 47). Strategic disclosure programs result in people making empowered decisions about whether to disclose their diagnosis or not (67). A strengths-based case management by a trained midwife, shared decision-making, and shared action plans during the perinatal period (i.e., from preconception to 1 year after childbirth) could improve self-agency, empowerment, and maternal and children's outcomes (68–70). Future studies should investigate the potential effectiveness of these interventions in ASD. In other conditions such as serious mental illness, mothers report concerns about passing on their condition to their children (71). This might also be the case for autistic mothers, although this should be further investigated. Psychoeducation and family psychoeducation (e.g., ASD and pregnancy-related outcomes, heredity, risk factors for perinatal depression and how to deal with them, early detection, and intervention of ASD in children) provided during the perinatal period could prevent perinatal depression and improve maternal and children's outcomes.

Psychiatric rehabilitation interventions on reproductive health should also discuss the centrality of motherhood for autistic women (or, in contrast, their self-reported “absence of maternal instinct”) (32) and its impact on a person's sense of personal identity.

#### Gender Differences in Treatment Effectiveness: Implications for Designing Interventions

Among the psychosocial interventions addressing romantic relationships or reproductive health in autistic people, no gender differences were reported on treatment outcomes after SST or psychosexual training (15, 17). Although it has been proposed that autistic women and men had similar needs for care and could benefit from the same interventions (15), autistic women may face unique challenges when subjective aspects (e.g., impact of gender-related social norms, roles, and expectations on a person's sense of identity) (32, 34) and specific domains (e.g., romantic relationships and reproductive health) (24, 26, 27, 34, 45) are considered.

While recent epidemiological studies have found lower male-to-female ratios than previously reported (18), the predominance of males in research samples could have affected both the development and the evaluation of psychosocial treatments (18, 19). It can be hypothesized that men, presumed to have poorer social function, are all referred to psychosocial treatment, whereas only the women with severe social communication impairments are referred (19). Another hypothesis could be that existing interventions (mostly developed in male samples) do not meet the unique needs for care of women and gender-diverse individuals (e.g., discussing gender variance and gender-related social norms, roles, and expectations, prevention of sexual abuse, and reproductive health) (4, 26, 44). The development of strengths-based, gender-sensitive (i.e., taking into account gender variance, autistic people's lived experience, and women's needs for care) interventions addressing romantic relationships and reproductive health is needed (4, 13).

## Limitations

There are some limitations to this review due to the heterogeneity in samples, in the methods, scales, interventions, and the reported outcomes. Few studies reported longitudinal outcomes, and only a small number of psychosocial treatments addressed romantic relationships or reproductive health from the perspective of autistic women or autistic gender-diverse individuals. The low–moderate quality of the included studies is also a limitation. This review excluded studies where women's outcomes in specific domains (i.e., romantic relationships and reproductive health) were not the main focus, which means that some needs for care in other domains (e.g., vocational function or leisure activities) might have been overlooked. However, by focusing on these domains using a broad definition of female gender (i.e., cisgender and gender-diverse individuals), this review provides a more accurate understanding of this population's needs for care relating to psychosocial treatments. The underreporting of negative or non-significant results due to publication bias from this review might have limited the accuracy of the synthesis.

In short, gender may influence a person's needs for care and treatment outcomes in autistic people, but this remains underinvestigated. Psychosocial treatments addressing romantic relationships or reproductive health remain underdeveloped and are often inadequate for women's and gender-diverse individuals' needs for care. High-quality research taking into account the perspectives and lived experiences of autistic women and gender-diverse individuals relating to their needs for care in romantic relationships and reproductive health is needed to guide the development of new interventions. Adopting a woman's health lens during the care of autistic women (e.g., with an evaluation and interventions by a trained midwife or by integrating gender-sensitive content in psychosocial treatments) might improve their access to physical healthcare services and to adequate support from perinatal services. This remains, however, to be investigated.

## Data Availability Statement

The original contributions presented in the study are included in the article/supplementary material, further inquiries can be directed to the corresponding author/s.

## Author Contributions

MD and JD carried out the literature review and drafted the article. Both authors contributed to and approved the final manuscript.

## Conflict of Interest

The authors declare that the research was conducted in the absence of any commercial or financial relationships that could be construed as a potential conflict of interest.

## References

[1] Hull L Petrides KV Mandy W. The female autism phenotype and camouflaging: a narrative review. Rev J Autism Dev Disord. (2020) 7:306–17. 10.3389/frym.2019.00129

[2] American Psychiatric Association. Diagnostic and Statistical Manual of Mental Disorders (DSM5), 5th ed. Washington, DC: American Psychiatric Press (2013). 10.1176/appi.books.9780890425596

[3] Cooper K Smith LGE Russell AJ. Gender identity in autism: sex differences in social affiliation with gender groups. J Autism Dev Disord. (2018) 48:3995–4006. 10.1007/s10803-018-3590-1 29705922 PMC6223803

[4] Strang JF van der Miesen AI Caplan R Hughes C daVanport S Lai MC. Both sex- and gender-related factors should be considered in autism research and clinical practice. Autism. (2020) 24:539–543. 10.1177/1362361320913192 32299242

[5] Farkas M Anthony WA. Psychiatric rehabilitation interventions: a review. Int Rev Psychiatry. (2010) 22:114–29. 10.3109/09540261003730372 20504052

[6] Franck N Bon L Dekerle M Plasse J Massoubre C Pommier R. Satisfaction and needs in serious mental illness and autism spectrum disorder: the REHABase psychosocial rehabilitation project. Psychiatr Serv. (2019) 70:316–23 10.1176/appi.ps.201800420 30691384

[7] Dubreucq J Plasse J Gabayet F Faraldo M Blanc O Chereau I. Self-stigma in serious mental illness and autism spectrum disorder: results from the REHABase national psychiatric rehabilitation cohort. Eur Psychiatry. (2020) 63:e13. 10.1192/j.eurpsy.2019.12 32093806 PMC7315867

[8] Dandil Y Smith K Kinnaird E Toloza C Tchanturia K. Cognitive remediation interventions in autism spectrum condition: a systematic review. Front Psychiatry. (2020) 11:722. 10.3389/fpsyt.2020.00722 32793009 PMC7393993

[9] Mavranezouli I Megnin-Viggars O Cheema N Howlin P Baron-Cohen S Pilling S. The cost-effectiveness of supported employment for adults with autism in the United Kingdom. Autism. (2014) 18:975–84. 10.1177/1362361313505720 24126866 PMC4230968

[10] Keefer A White SW Vasa RA Reaven J. Psychosocial interventions for internalizing disorders in youth and adults with ASD. Int Rev Psychiatry. (2018) 30:62–77. 10.1080/09540261.2018.1432575 29537895

[11] Spain D Blainey SH. Group social skills interventions for adults with high-functioning autism spectrum disorders: a systematic review. Autism. (2015) 19:874–86. 10.1177/1362361315587659 26045543

[12] Hillier A Campbell H Mastriani K Izzo MV Kool-Tukcer AK Cherry L. Two-year evaluation of a vocational support program for adults on the autism spectrum. Career Dev Transit Except Individ. (2007) 30:35–47 10.1177/08857288070300010501

[13] DaWalt LS Taylor JL Bishop S Hall LJ Steinbrenner JD Kraemer B. Sex differences in social participation of high school students with autism spectrum disorder. Autism Res. (2020) 13:2155–63. 10.1002/aur.2348 32881417 PMC7749043

[14] Taylor JL Henninger NA Mailick MR. Longitudinal patterns of employment and postsecondary education for adults with autism and average-range IQ. Autism. (2015) 19:785–93. 10.1177/1362361315585643 26019306 PMC4581899

[15] McVey AJ Schiltz H Haendel A Dolan BK Willar KS Pleiss S. Brief report: does gender matter in intervention for ASD? Examining the impact of the PEERS® social skills intervention on social behavior among females with ASD. J Autism Dev Disord. (2017) 47:2282–9. 10.1007/s10803-017-3121-5 28391452 PMC6419962

[16] Sung C Sánchez J Kuo HJ Wang CC Leahy MJ. Gender differences in vocational rehabilitation service predictors of successful competitive employment for transition-aged individuals with autism. J Autism Dev Disord. (2015) 45:3204–18. 10.1007/s10803-015-2480-z 26060047

[17] Visser K Greaves-Lord K Tick NT Verhulst FC Maras A van der Vegt EJM. A randomized controlled trial to examine the effects of the Tackling Teenage psychosexual training program for adolescents with autism spectrum disorder. J Child Psychol Psychiatry. (2017) 58:840–50. 10.1111/jcpp.12709 28276079

[18] Lai MC Lombardo MV Auyeung B Chakrabarti B Baron-Cohen S. Sex/gender differences and autism: setting the scene for future research. J Am Acad Child Adolesc Psychiatry. (2015) 54:11–24. 10.1016/j.jaac.2014.10.003 25524786 PMC4284309

[19] Dubreucq J Haesebaert F Plasse J Dubreucq M Franck N. A systematic review and meta-analysis of social skills training for adults with Autism Spectrum Disorder. J Autism Dev Disord. Revised. 10.1007/s10803-021-05058-w 33963965

[20] Laugeson EA Gantman A Kapp SK Orenski K Ellingsen R. A Randomized controlled trial to improve social skills in young adults with autism spectrum disorder: the UCLA PEERS(®) program. J Autism Dev Disord. (2015) 45:3978–89. 10.1007/s10803-015-2504-8 26109247

[21] Cunningham A Sperry L Brady MP Peluso PR Pauletti RE. The effects of a romantic relationship treatment option for adults with autism spectrum disorder. Counsel Outcome Res Eval. (2016) 7:99–110. 10.1177/2150137816668561

[22] Tint A Weiss JA. A qualitative study of the service experiences of women with autism spectrum disorder. Autism. (2018) 22:928–37. 10.1177/1362361317702561 28914071

[23] Urbano MR Hartmann K Deutsch SI Bondi Polychronopoulos GM Dorbin V. Relationships, sexuality, and intimacy in autism spectrum disorders. In: Fitzgerald M, editor. Recent Advances in Autism Spectrum Disorders - Volume I. IntechOpen (2013). Available online at: https://www.intechopen.com/books/recent-advances-in-autism-spectrum-disorders-volume-i/relationships-sexuality-and-intimacy-in-autism-spectrum-disorders (accessed January 12, 2021).

[24] Pecora LA Hancock GI Mesibov GB Stokes MA. Characterising the sexuality and sexual experiences of autistic females. J Autism Dev Disord. (2019) 49:4834–46. 10.1007/s10803-019-04204-9 31463632

[25] George R Stokes MA. A quantitative analysis of mental health among sexual and gender minority groups in ASD. J Autism Dev Disord. (2018) 48:2052–63. 10.1007/s10803-018-3469-1 29362955

[26] Taylor JL DaWalt LS. Working toward a better understanding of the life experiences of women on the autism spectrum. Autism. (2020) 24:1027–30. 10.1177/1362361320913754 32564668 PMC12035819

[27] Pohl AL Crockford SK Blakemore M Allison C Baron-Cohen S. A comparative study of autistic and non-autistic women's experience of motherhood. Mol Autism. (2020) 11:3. 10.1186/s13229-019-0304-2 31911826 PMC6945630

[28] Moher D Shamseer L Clarke M Ghersi D Liberati A Petticrew M. Preferred reporting items for systematic review and meta-analysis protocols (PRISMA-P) 2015 statement. Syst Rev. (2015) 4:1. 10.1186/2046-4053-4-1 25554246 PMC4320440

[29] Pluye P Robert E Cargo M Bartlett G O'Cathain A Griffiths F. Proposal: A Mixed Methods Appraisal Tool for Systematic Mixed Studies Reviews. (2011). Available online at: http://mixedmethodsappraisaltoolpublic.pbworks.com. Archived by WebCite® at http://www.webcitation.org/5tTRTc9yJ (accessed October 4, 2018).

[30] Jamison TR Schuttler JO. Overview and preliminary evidence for a social skills and self-care curriculum for adolescent females with autism: the girls night out model. J Autism Dev Disord. (2017) 47:110–25. 10.1007/s10803-016-2939-6 27757738

[31] Sundelin HE Stephansson O Hultman CM Ludvigsson JF. Pregnancy outcomes in women with autism: a nationwide population-based cohort study. Clin Epidemiol. (2018) 10:1817–1826. 10.2147/CLEP.S176910 30555264 PMC6280895

[32] Kanfiszer L Davies F Collins S. 'I was just so different': the experiences of women diagnosed with an autism spectrum disorder in adulthood in relation to gender and social relationships. Autism. (2017) 21:661–9. 10.1177/1362361316687987 28326792

[33] Sala G Hooley M Stokes MA. Romantic intimacy in autism: a qualitative analysis. J Autism Dev Disord. (2020) 50:4133–47. 10.1007/s10803-020-04377-8 31993919

[34] Bargiela S. Steward of late-diagnosed women with autism spectrum conditions: an investigation of the female autism phenotype. J Autism Dev Disord. (2016) 46:3281–94. 10.1007/s10803-016-2872-8 27457364 PMC5040731

[35] Bejerot S Eriksson JM. Sexuality and gender role in autism spectrum disorder: a case control study. PLoS ONE. (2014) 9:e87961. 10.1371/journal.pone.0087961 24498228 PMC3909328

[36] Busch HH. Dimensions of sexuality among young women, with and without autism, with predominantly sexual minority identities. Sex Disabil. (2018) 37:275–92. 10.1007/s11195-018-9532-1

[37] Byers ES Nichols S Voyer SD Reilly G. Sexual well-being of a community sample of high-functioning adults on the autism spectrum who have been in a romantic relationship. Autism. (2013) 17:418–33. 10.1177/1362361311431950 23045223

[38] Strang JF Knauss M van der Miesen A McGuire JK Kenworthy L Caplan R. A clinical program for transgender and gender-diverse neurodiverse/autistic adolescents developed through community-based participatory design. J Clin Child Adolesc Psychol. (2020) 6:1–16. 10.1080/15374416.2020.1731817 32375521 PMC11362985

[39] Baldwin S Costley D. The experiences and needs of female adults with high-functioning autism spectrum disorder. Autism. (2016) 20:483–95. 10.1177/1362361315590805 26111537

[40] Strunz S Schermuck C Ballerstein S Ahlers CJ Dziobek I Roepke S. Romantic relationships and relationship satisfaction among adults with asperger syndrome and high-functioning autism. J Clin Psychol. (2017) 73:113–25. 10.1002/jclp.22319 27196958

[41] Smith R Netto J Gribble NC Falkmer M. 'At the end of the day, it's love': an exploration of relationships in neurodiverse couples. J Autism Dev Disord. (2020). 10.1007/s10803-020-04790-z. [Epub ahead of print]. 33216278

[42] Corona LL Fox SA Christodulu KV Worlock JA. Providing education on sexuality and relationships to adolescents with autism spectrum disorder and their parents. Sex Disabil. (2015) 34:199–214. 10.1007/s11195-015-9424-6

[43] Hénault I. Programme d'éducation sociosexuelle. In: Hénault I, editor. Sexualité et Syndrome d'Asperger, Éducation Sexuelle et Interventions Auprès de la Personne Autiste. Louvain-la-Neuve: De Boeck (2010), p. 115–20.

[44] Dubreucq M Dubreucq J. Recovery-oriented program on sexuality and prevention of sexual abuse for women with Autism Spectrum Disorder (ASD): a new intervention based on a qualitative analysis. In preparation.

[45] Pecora LA Hancock GI Hooley M Demmer DH Attwood T Mesibov GB. Gender identity, sexual orientation and adverse sexual experiences in autistic females. Mol Autism. (2020) 11:57. 10.1186/s13229-020-00363-0 32653016 PMC7353794

[46] Gardner M Suplee PD Bloch J Lecks K. Exploratory study of childbearing experiences of women with asperger syndrome. Nurs Womens Health. (2016) 20:28–37. 10.1016/j.nwh.2015.12.001 26902438

[47] Rogers C Lepherd L Ganguly R Jacob-Rogers S. Perinatal issues for women with high functioning autism spectrum disorder. Women Birth. (2017) 30:e89–95. 10.1016/j.wombi.2016.09.009 27751685

[48] Donovan J. Childbirth experiences of women with autism spectrum disorder in an acute care setting. Nurs Womens Health. (2020) 24:165–74. 10.1016/j.nwh.2020.04.001 32389581

[49] Kassee C Babinski S Tint A Lunsky Y Brown HK Ameis SH. Physical health of autistic girls and women: a scoping review. Mol Autism. (2020) 11:84. 10.1186/s13229-020-00380-z 33109257 PMC7590704

[50] Ames JL Massolo ML Davignon MN Qian Y Croen LA. Healthcare service utilization and cost among transition-age youth with autism spectrum disorder and other special healthcare needs. Autism. (2020). 10.1177/1362361320931268. [Epub ahead of print]. 32583679

[51] Milner V McIntosh H Colvert E Happé F. A qualitative exploration of the female experience of autism spectrum disorder (ASD). J Autism Dev Disord. (2019) 49:2389–402. 10.1007/s10803-019-03906-4 30790191 PMC6546643

[52] Sedgewick F Hill V Pellicano E. 'It's different for girls': gender differences in the friendships and conflict of autistic and neurotypical adolescents. Autism. (2019) 23:1119–32. 10.1177/1362361318794930 30280923

[53] Cage E Troxell-Whitman Z. Understanding the reasons, contexts and costs of camouflaging for autistic adults. J Autism Dev Disord. (2019) 49:1899–911. 10.1007/s10803-018-03878-x 30627892 PMC6483965

[54] Moody CT Laugeson EA. Social skills training in autism spectrum disorder across the lifespan. Child Adolesc Psychiatr Clin N Am. (2020) 29:359–71. 10.1016/j.chc.2019.11.001 32169267

[55] Livingston LA Happé F. Conceptualising compensation in neurodevelopmental disorders: Reflections from autism spectrum disorder. Neurosci Biobehav Rev. (2017) 80:729–42. 10.1016/j.neubiorev.2017.06.005 28642070 PMC7374933

[56] Livingston LA Colvert E Social Relationships Study Team Bolton P Happé F. Good social skills despite poor theory of mind: exploring compensation in autism spectrum disorder. J Child Psychol Psychiatry. (2019) 60:102–10. 10.1111/jcpp.12886 29582425 PMC6334505

[57] Livingston LA Shah P Happé F. Compensatory strategies below the behavioural surface in autism: a qualitative study. Lancet Psychiatry. (2019) 6:766–77. 10.1016/S2215-0366(19)30224-X 31350208 PMC6706698

[58] Cassidy SA Gould K Townsend E Pelton M Robertson AE Rodgers J. Is camouflaging autistic traits associated with suicidal thoughts and behaviours? Expanding the interpersonal psychological theory of suicide in an undergraduate student sample. J Autism Dev Disord. (2020) 50:3638–48. 10.1007/s10803-019-04323-3 31820344 PMC7502035

[59] Tint A Hamdani Y Sawyer A Desarkar P Ameis SH Bardikoff N. Wellness efforts for autistic women. Curr Dev Disord Rep. (2018) 5:207–16. 10.1007/s40474-018-0148-z

[60] Schneid I Raz AE. The mask of autism: social camouflaging and impression management as coping/normalization from the perspectives of autistic adults. Soc Sci Med. (2020) 248:112826. 10.1016/j.socscimed.2020.112826 32036269

[61] Dubreucq J Plasse J Franck N. Self-stigma in serious mental illness: a systematic review of frequency, correlates, and consequences. Schizophr Bull. (2021). 10.1093/schbul/sbaa181. [Epub ahead of print]. 33459793 PMC8563656

[62] Bachmann CJ Höfer J Kamp-Becker I Küpper C Poustka L Roepke S. Internalised stigma in adults with autism: a German multi-center survey. Psychiatry Res. (2019) 276:94–99. 10.1016/j.psychres.2019.04.023 31030006

[63] Turner D Briken P Schöttle D. Sexual dysfunctions and their association with the dual control model of sexual response in men and women with high-functioning autism. J Clin Med. (2019) 8:425. 10.3390/jcm8040425 30925683 PMC6518023

[64] Barnett JP Maticka-Tyndale E. Qualitative exploration of sexual experiences among adults on the autism spectrum: implications for sex education. Perspect Sex Reprod Health. (2015) 47:171–9. 10.1363/47e5715 26418175

[65] Yanos PT Lysaker PH Silverstein SM Vayshenker B Gonzales L West ML. A randomized-controlled trial of treatment for self-stigma among persons diagnosed with schizophrenia-spectrum disorders. Soc Psychiatry Psychiatr Epidemiol. (2019) 54:1363–78. 10.1007/s00127-019-01702-0 30937510 PMC6773531

[66] Nagib W Wilton R. Gender matters in career exploration and job-seeking among adults with autism spectrum disorder: evidence from an online community. Disabil Rehabil. (2020) 42:2530–41. 10.1080/09638288.2019.1573936 30963797

[67] Mulfinger N Müller S Böge I Sakar V Corrigan PW Evans-Lacko S. Honest, open, proud for adolescents with mental illness: pilot randomized controlled trial. J Child Psychol Psychiatry. (2018) 59:684–91. 10.1111/jcpp.12853 29205343

[68] Hauck Y Rock D Jackiewicz T Jablensky A. Healthy babies for mothers with serious mental illness: a case management framework for mental health clinicians. Int J Ment Health Nurs. (2008) 17:383–91. 10.1111/j.1447-0349.2008.00573.x 19128285

[69] Galletly C Castle D Dark F Humberstone V Jablensky A Killackey E. Royal Australian and New Zealand college of psychiatrists clinical practice guidelines for the management of schizophrenia and related disorders. Aust N Z J Psychiatry. (2016) 50:410–72. 10.1177/0004867416641195 27106681

[70] Dubreucq M Provasi A Baudrant M Dubreucq J. Development of a shared decision-making tool from preconception to post-partum care for women with serious mental illness: a qualitative analysis. In preparation.

[71] Dolman C Jones IR Howard LM. Women with bipolar disorder and pregnancy: factors influencing their decision-making. BJPsych Open. (2016) 2:294–300. 10.1192/bjpo.bp.116.003079 27703792 PMC5013258

